# Colorectal Cancer Risk in Women with Gynecologic Cancers—A Population Retrospective Cohort Study

**DOI:** 10.3390/jcm10143127

**Published:** 2021-07-15

**Authors:** Szu-Chia Liao, Hong-Zen Yeh, Chi-Sen Chang, Wei-Chih Chen, Chih-Hsin Muo, Fung-Chang Sung

**Affiliations:** 1 Division of Gastroenterology, Department of Internal Medicine, Taichung Veterans General Hospital, Taichung 407, Taiwan; b8401084@gmail.com (S.-C.L.); hzen.yeh@gmail.com (H.-Z.Y.); changcs@vghtc.gov.tw (C.-S.C.); 2 Department of Public Health, China Medical University, Taichung 404, Taiwan; 3 Department of Internal Medicine, National Yang-Ming University, Taipei 112, Taiwan; 4 Department of Obstetrics and Gynecology, Taichung Veterans General Hospital, Taichung 407, Taiwan; awe@vghtc.gov.tw; 5 Management Office for Health Data, China Medical University Hospital, Taichung 404, Taiwan; b8507006@gmail.com; 6 Department of Health Services Administration, China Medical University, Taichung 404, Taiwan; 7 Department of Food Nutrition and Health Biotechnology, Asia University, Taichung 413, Taiwan

**Keywords:** colorectal cancer, gynecologic cancer, retrospective cohort study, colonoscopy screening

## Abstract

We conducted a retrospective cohort study to evaluate the subsequent colorectal cancer (CRC) risk for women with gynecologic malignancy using insurance claims data of Taiwan. We identified patients who survived cervical cancer (N = 25,370), endometrial cancer (N = 8149) and ovarian cancer (N = 7933) newly diagnosed from 1998 to 2010, and randomly selected comparisons (N = 165,808) without cancer, matched by age and diagnosis date. By the end of 2011, the incidence and hazard ratio (HR) of CRC were estimated. We found that CRC incidence rates were 1.26-, 2.20-, and 1.61-fold higher in women with cervical, endometrial and ovarian cancers, respectively, than in comparisons (1.09/1000 person–years). The CRC incidence increased with age. Higher adjusted HRs of CRC appeared within 3 years for women with endometrial and ovarian cancers, but not until the 4th to 7th years of follow up for cervical cancer survivals. Cancer treatments could reduce CRC risks, but not significantly. However, ovarian cancer patients receiving surgery alone had an incidence of 3.33/1000 person–years for CRC with an adjusted HR of 3.79 (95% CI 1.11–12.9) compared to patients without any treatment. In conclusion, gynecologic cancer patients are at an increased risk of developing CRC, sooner for those with endometrial or ovarian cancer than those with cervical cancer.

## 1. Introduction

Cervical, endometrial and ovarian cancers are gynecologic (GYN) cancers among the ten leading causes of deaths from cancer for women. Cervical cancer is the most common female cancer in developing countries and the eighth most common in the US women [1,2,3]. The prevalence of endometrial cancer is on the rise in developed countries, with the incidence higher than that of cervical cancer. Ovarian cancer is the second-most common cancer in women, with a higher incidence in developed countries. The 5-year survival rates of GYN cancers have improved over the past few decades due to the improved treatments [1]. GYN cancer survivors are at risk for a second cancer [4,5,6,7,8,9,10]. Human papillomavirus infection, smoking, obesity, hormone replacement therapy, radiotherapy and hereditary nonpolyposis colorectal cancer (HNPCC) are associated with a secondary malignancy [6,10,11]. A meta-analysis found that the standardized incidence ratios (SIRs) on all types of second cancer risk ranged from 1.0 to 1.4 for women with primary breast cancer, with the risk greater for women of less than 50 years than those who were older (SIR 1.51 vs. 1.11) [11].

Colorectal cancer (CRC) has become the second or third leading cause in cancer-related deaths in women [12,13,14,15]. GYN cancers and CRC share some common risk factors, such as obesity, lifestyle and socioeconomic status [10,11,12,14,16]. Thus, the risk of CRC development is an important concern for women with GYN cancer. Previous epidemiologic studies have shown conflicting results about the CRC risk in women with prior cervical or endometrial cancers [4,17,18,19,20,21,22]. Studies on the risk of subsequent CRC after radiotherapy for cervical cancer have conflicting results. Women with previous endometrial or ovarian cancer with or without radiotherapy have been found to be at increased risk for CRC [14,22].

Using cancer registries in European countries and the United States, Chaturvedi et al. followed 104,760 one-year survivors of cervical cancer for 40 years [19]. Patients treated with heavy radiotherapy have a higher SIR for second cancers, including colorectal cancer and other GYN cancers. Limited data are available on the risk of CRC for Asian women with GYN. A retrospective study followed 52,972 women with cervical cancer for 9 years using the Taiwan Cancer Registry and found the second cancer risk was greater for rectal cancer than for colon cancer (SIR = 1.31 vs. 1.13) [5]. The effects of treatment for cervical cancer on the risk of CRC have not been clarified in the study.

No study has compared the CRC risk for women with GYN cancers by treatment modality other than with or without radiotherapy. In the present study, we established cohorts of survivors with major GYN cancers, including cervical, endometrial and ovarian cancers, to evaluate the risk of subsequent CRC. Risks of the second CRC cancers were also assessed for patients with GYN treatment methods.

## 2. Methods and Materials

### 2.1. Study Design, Data Source and Study Subjects

We performed a population-based retrospective cohort study using data obtained from Taiwan National Health Insurance, which is a universal health insurance system with over 99% of the population covered. We used 1998–2011 claims data, which included inpatient and outpatient records for cancer care and a registry for catastrophic illnesses. The International Classification of Diseases, 9th Revision, Clinical Modification (ICD-9-CM) and A-code was applied to retrieve information on diagnosis. 

From the registry for catastrophic illnesses, we identified 41,452 cases of GYN cancers with at least one-year survival from 1998 to 2010, for the study cohorts. Patients with the history of CRC at the baseline were excluded. The GYN cancer cohort included 25,370 cases of cervical cancer (ICD-9-CM code 180), 8149 cases of endometrial cancer (ICD-9-CM code 182) and 7933 cases of ovarian cancer (ICD-9-CM code 183). The diagnosis date was designated as index date. Using a ratio of 1:4, 165,808 women free from any cancer were randomly selected as the reference cohort, and frequency matched with all GYN cases by age and index date. Follow up began 1 year after the subject was included in the cohort until the date of CRC diagnosis or the end of 2011, whichever occurred first. Subjects lost to follow up were censored. Subsequent CRC cases were identified by linkage within the respective cancer registry files and confirmed by the registry for catastrophic illnesses.

### 2.2. Statistical Analysis

Data analysis first displayed sociodemographic characteristics (age and occupation) and comorbidities among cohorts. Comorbidities included diabetes mellitus (ICD-9-CM 250), hypertension (ICD-9-CM 401–405,997.91), hyperlipidemia (ICD-9-CM 272), non-infectious enteritis and colitis (ICD-9-CM 555–558), anal and rectal polyp (ICD-9-CM 560.9), benign neoplasm of the colon (ICD-9-CM 211.3), and cholecystectomy (ICD-9-CM 51.22–51.23) [23]. Distributions of age (30–39, 40–49, 50–64, and >60 years), occupation and comorbidities were compared between the GYN cohorts and reference cohort and examined using a Chi-square test for categorical variables and a *t*-test for continuous variables. We calculated the incidence rates of subsequent CRC for each cohort during the follow-up period. The Cox proportional hazards regression analysis was used to estimate the hazard ratios (HRs) and 95% confidence intervals (CIs) of CRC associated with GYN cancers and treatment modalities. The multivariable Cox model was used to calculate adjusted HR (aHR) controlling for demographic factors and comorbidities. To assess the effect of cancer therapy, GYN cohorts were stratified into five groups by therapeutic modalities: radiation therapy (RT) only, chemotherapy (CT) only, combination of RT and CT (RT/CT), surgery only and no treatment (non-RT/CT/surgery). We used the no treatment group as a reference to examine whether RT, CT, surgery, and RT/CT were associated the CRC risk. We also calculated the HRs of CRC by the follow-up duration, <1, 2–3, 4–5, 6–7, 8–9 and ≥10 years for the GYN cohorts. In order to evaluate the competing risk of death, we also used the sub-distribution model to estimate the overall sub-hazard ratio (SHR) of CRC cancer associated with each of the 3 GYN cancers. All data analyses were performed using the SAS 9.3 statistical package (SAS Institute Inc., Cary, NC, USA). The study was approved by Research Ethics Committee at China Medical University and Hospital (CMUH104-REC2-115).

## 3. Results

All GYN cancer cases and the reference cohort were similar in distributions of age, with the mean age of 54.9 years (Table 1). Patients with cervical cancer were older than patients with endometrial and ovarian cancers (means 56.2, 53.2 and 52.0 years, respectively). Women with endometrial cancer and ovarian cancer were more likely to work in white collar jobs. Overall, GYN cancer patients were more prevalent than the reference cohort with diabetes mellitus, hypertension, non-infectious enteritis and colitis, anal and rectal polyps, and benign neoplasm of the colon. The prevalence rates of hyperlipidemia and cholecystectomy were similar.

The overall CRC incidence rate was the highest in the endometrial cohort, followed by the ovarian cohort and cervical cohort (2.20, 1.76 and 1.37 per 1000 person–years, respectively) with aHRs of 2.26 (95% confidence interval (CI): 1.77–2.90), 2.09 (95% CI: 1.59–2.76) and 1.20 (95% CI: 1.03–1.40), compared to the reference cohort (1.09 per 10,000 person–years) (Table 2). The age-specific CRC cancer incidence increased with age in each cohort. However, the age-specific HR of CRC, relative to the reference cohort, decreased with age, particularly for patients with endometrial and ovarian cancer. We further used the sub-distribution model to estimate the adjusted hazard ratio (aSHR) of CRC associated with the competing risk of death in women with these GYN cancers. The overall aSHRs of developing CRC were 1.04 (95% CI: 0.89–1.21), 1.97 (95% CI: 1.54–2.52) and 1.53 (95% CI: 1.16–2.01), respectively, in women with cervical, endometrial and ovarian cancers.

Table 3 shows the CRC risk associated with treatment modalities. The incidence rates were higher in patients with cervical cancer and endometrial cancer receiving no treatment (1.90 and 3.84 per 1000 person–years, respectively) than those with treatment. The aHR was significant for those with endometrial cancer (aHR = 3.38, 95% CI: 1.61–7.11), compared to the reference cohort, but not significant for those with cervical cancer. Treatments reduced the CRC incidence rates in both cohorts, with significant aHRs in the endometrial cancer cohort but not significant in the cervical cancer cohort. However, all reduced aHRs were not significant for patients with treatments, compared to those with no treatment. On the other hand, the CRC incidence rate in ovarian cancer patients was 4.6-fold greater in those undergoing surgery than those receiving no treatment (3.33 vs. 0.73 per 1000 person–years), with an aHR of 3.56 (95% CI: 2.23–5.68) compared with controls. Most ovarian cancer patients received chemotherapy (5069/7933) and had an adjusted HR of 1.95 (95% CI: 1.35–2.80). 

**Figure 1:**
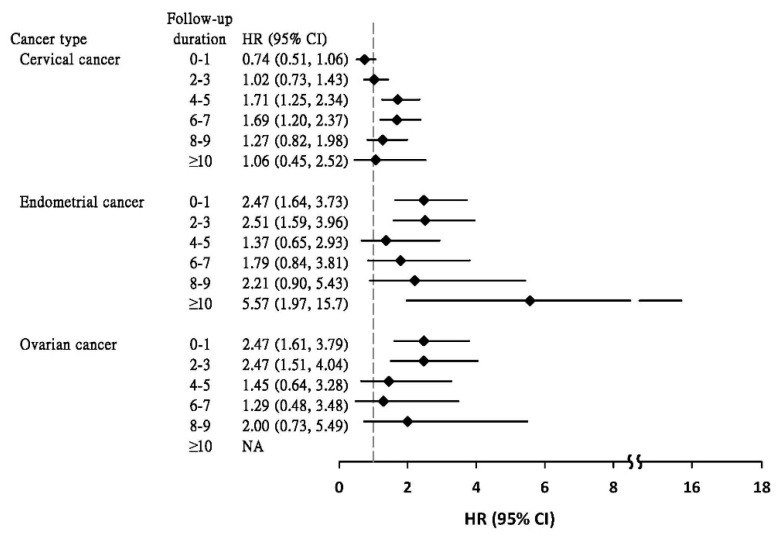
Cox proportional hazards regression analysis estimated adjusted hazard ratio of colorectal cancer for patients with gynecologic cancers compared to reference cohort by follow-up year.

Figure 1 shows the Cox model-estimated aHRs of CRC for GYN cohorts in a 10-year follow-up period, compared with the reference cohort. The incident CRC developed earlier in women with the endometrial cohort and the ovarian cohort than in women with cervical cancer. Elevated aHRs were significant within the first 3 years of follow up for women with endometrial cancer and ovarian cancer, but not until 4th to 7th years for women with cervical cancer.

**Figure 2:**
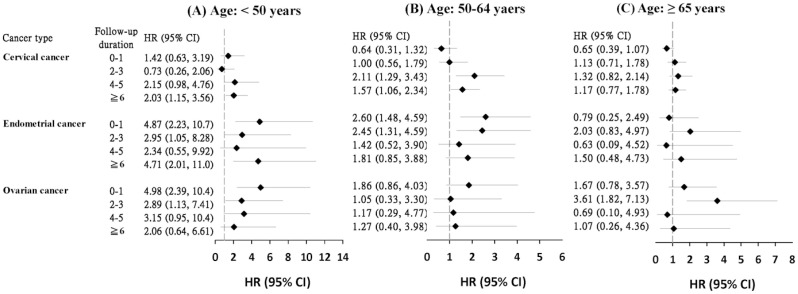
Cox proportional hazards regression analysis estimated adjusted hazard ratios of colorectal cancer for patients with gynecologic cancers relative to comparison cohort by follow-up year and age group. (**A**) Age: <50 years, (**B**) Age: 50–64 years, (**C**) Age: ≥65 years

Figure 2 shows the Cox model estimated age-specific aHRs of CRC during the follow-up period. The hazards of developing CRC were all greater for younger GYN patients, particularly during the first 3 years of follow up for women <50 years old with endometrial cancer and ovarian cancer.

## 4. Discussion

This population-based retrospective cohort study showed that women with major GYN cancers are at an elevated risk of developing CRC. The CRC risk is the highest for women with endometrial cancer, followed by ovarian cancer and cervical cancer. The CRC risk varied not only by GYN cancer type, but also by the follow-up period, cancer treatment modality and age. Previous studies on relationships between a second CRC and GYN cancers are inconsistent [4,17,18,19,20,21,22]. In general, the CRC risks found were stronger for patients with ovarian cancer and endometrial cancer than for patients with cervical cancer. Weaker relationships between CRC and cervical cancer in these studies are consistent with our findings. We failed to identify the CRC risk in association with treatment modality in cervical cancer.

A retrospective cohort study using the US cancer registry data of the Surveillance, Epidemiology, and End Results (SEER) program found women with GYN cancer tended to have a higher CRC incidence in the first 6 months after the diagnosis of the cancer; the estimated SIR of subsequent CRC is significant for those with ovarian cancer (SIR: 2.20, 95% CI: 1.06–2.58), but not for endometrial cancer [22]. Another study using SEER data found the risk of CRC was the highest in 12–24 months after the diagnosis of endometrial cancer [14]. The Swedish record-linkage study also found a significant SIR of 1.64 (95% CI: 1.24–2.11) for CRC within 2 years for women with ovarian cancer [4]. 

The exact mechanisms associated with CRC risk among women with GYN cancers remain unclear. GYN cancers shared the same risk factors with CRC, including hormone modulation, lifestyle and hereditary diseases. Decreased exposure to estrogen may protect against colorectal, endometrial, and ovarian cancer [24]. Nulliparous women and women using hormone replacement therapy are at a high risk [16]. Dietary factors and obesity are the shared risk factors in colorectal and ovarian or endometrial cancer [14]. Estrogen levels are elevated in obese persons [25]. In addition, the familial CRC syndrome of HNPCC can appear in the early development of colorectal, endometrial and ovarian cancer [26]. However, HNPCC is not prevalent in our population. HNPCC may do little to explain the association between GYN cancers and CRC.

GYN cancer detection and treatment may in part explain the CRC risk variations among GYN cancers. The latent periods of subsequent CRC for women with cervical cancer is longer than that for women with endometrial cancer and ovarian cancer. The pap test helps to detect cervical cancer in the early stages. The detection and treatment of carcinoma in situ of the cervix may prevent not only the development of an invasive carcinoma of the cervix but also other cancer. On the other hand, ovarian cancer and endometrial cancer are more likely not detected until they are in more advanced stages. This may also explain in part why the incidence of CRC in cervical cancer patients was lower than that in ovarian cancer and endometrial cancer patients. 

Some women may have developed CRC by the time they are diagnosed with ovarian cancer and endometrial cancer. In our study, the CRC incidence was the highest in women receiving surgery alone for ovarian cancer treatment. These patients might have received more screening modalities. A higher CRC incidence is thus identified in a shorter follow-up time for ovarian cancer patients than for cervical cancer patients. Most patients with endometrial cancer received surgery alone, but they had the lowest CRC incidence. These patients might have the disease diagnosed at an advanced stage. This is probably why CRC incidence was the highest in endometrial cancer patients receiving no treatment. On the other hand, the incidence among ovarian cancer patients was the lowest for those receiving no treatment. However, there were few ovarian cases receiving no treatment. A further investigation with a more ovarian cases is needed to address the finding.

Evidence from previous studies has shown the risk of CRC is elevated for cervical cancer patients after receiving RT [18,19]. Brown et al. found the RT treatment increased colon cancer risk after endometrial cancer [18]. In the present study, the CRC risk increased after RT for endometrial cancer and ovarian cancer, but not for cervical cancer. The relationship between CRC risk and endometrial cancers treated with RT in our study is compatible with findings in two studies using the US Survival, Epidemiology, and End Results database [18,20]. 

In our study, 70.7% patients with ovarian cancer, 10.1% patients with endometrial cancer and 6.09% patients with cervical cancer received CT. The subsequent CRC risk after CT was significant for those with endometrial cancer and ovarian cancer. No previous study has observed the CRC risk for GYN cancers after CT. Further data analysis showed that the age-specific GYN cancer cohort to the reference cohort risk of CRC was greater for younger patients than older patients after CT. As for 30–49 years old patients, the adjusted HRs of CRC were 5.44 (95% CI: 2.23–13.3) for those with endometrial cancer and 3.29 (95% CI: 1.86–5.84) for those with ovarian cancer. In general, younger women might have these cancers diagnosed at an earlier stage and have longer survival than older women have. Longer survival increases the detection of CRC cancer. The greater impact of CT for young GYN patients than older patients could be true, because of low CRC incidence in younger general population [27]. A further investigation for the impact of CT regimens needs to be addressed. Routine gynecologic examination and cancer screening are also recommended for these younger women.

Boice et al. found the risk of secondary cancer was greater for young GYN patients after RT [28]. A previous study on testicular cancer patients noted that platinum-based chemotherapy had induced leukemia and solid organ tumors, including colon cancer [15,29,30,31]. Travis et al. found a higher risk of leukemia in ovarian cancer patients after receiving CT [32], but no report on the risk of CRC. No other report has addressed the carcinogenic effect after CT for endometrial and ovarian cancers. We suspect that harder follow-up checks for GYN cancer patients may also explain in part the increased identification of CRC.

Our study results should be interpreted with caution because of limitations. First, data on patient lifestyles and family history of diseases were not adjusted in data analyses because the information is not available from the NHRI records. Second, the NHRI records also provide no information on cancer stage and dosages of CT and RT, and we are unable to measure the dose–response association between treatment and CRC risk. Third, cancer patients covered in the insurance system are registered in the catastrophic illnesses group eligible for treatment benefit with discounted treatment costs. The insurance system provides no guides on which treatment modalities are usually used on treatment by the cancer stage. Our study could not differentiate whether the health insurance policies affect the development of CRC. However, further study is needed to investigate factors associated with increased CRC risk in women receiving surgery for ovarian cancer. Information on images of colonoscopy screening is also unavailable, and we are unable to prove whether hard follow-up checkups increase the diagnosis of CRC for GYN patients. However, all cancer patients have been registered as catastrophic illnesses, and the misdiagnosis of GYN cancer and CRC is unlikely in this study.

## 5. Conclusions

In this study, the number of patients with cervical cancer was much greater than that of endometrial and ovarian cancers. The risk of developing a second CRC was higher for patients with endometrial and ovarian cancers than for those with cervical cancer. The younger patients were at a higher impact after treatment. The risk of developing CRC after GYN cancer therapy is an important concern, because the CRC risk varied by the cancer treatment method among GYN cancers. The elevated incidence of CRC associated with surgery in patients with endometrial and ovarian cancers, but not cervical cancer should prompt the mechanism investigation. Colonoscopy screening for the subsequent development of CRC in these GYN cancer patients should be performed as soon as possible, especially for patients below 50 years old, in the early years after cancer diagnosis and after ever receiving chemotherapy or radiotherapy.

## Figures and Tables

**Table 1:** Distributions of gender, age, and comorbidity among gynecologic cancer cohorts and reference cohort identified from 1998 to 2010.

	Cancer Cohorts				Reference	*p* Value
	Cervical	Endometrial	Ovarian	Total		
Total population, n (%)	25,370 (61.2)	8149 (19.7)	7933 (19.1)	41,452 (100)	165,808 (100)	
Age, n (%)						
30–39	2905 (11.5)	770 (9.45)	1284 (16.2)	4959 (12.0)	19,836 (12.0)	0.95
40–49	6727 (26.5)	2293 (28.1)	2514 (31.7)	11,534 (27.8)	46,136 (27.8)	
50–64	8513 (33.6)	3914 (48.0)	2845 (35.9)	15,272 (36.8)	61,088 (36.8)	
≥65	7225 (28.5)	1172 (14.4)	1290 (16.3)	9687 (23.4)	38,748 (23.4)	
Mean (SD)	56.2 (13.5)	53.6 (10.7)	52.0 (12.1)	54.9 (12.8)	54.8 (12.9)	0.36
Occupation n, (%)						
White collar	11,034 (43.5)	4495 (55.2)	4421 (55.7)	19,950 (48.1)	81,365 (49.1)	<0.0001
Blue collar	11,902 (46.9)	2963 (36.4)	2805 (35.4)	17,670 (42.6)	71,622 (43.2)	
Others	2405 (9.48)	651 (8.36)	693 (8.74)	3779 (9.12)	12,634 (7.62)	
Missing	29 (0.11)	10 (0.12)	14 (0.18)	53 (0.13)	187 (0.11)	
Comorbidity, n (%)						
Diabetes	3139 (12.4)	1325 (16.3)	794 (10.0)	5258 (12.7)	18,894 (11.4)	<0.0001
Hypertension	2938 (11.6)	1180 (14.5)	848 (10.7)	4966 (12.0)	12,275 (7.40)	<0.0001
Hyperlipidemia	520 (2.05)	187 (2.29)	143 (1.80)	850 (2.05)	3366 (2.03)	0.79
Non-infectious enteritis and colitis	419 (1.65)	70 (0.86)	86 (1.08)	575 (1.39)	1944 (1.17)	0.0004
Anal and rectal polyp	25 (0.10)	0 (0.00)	6 (0.08)	31 (0.07)	56 (0.03)	0.0003
Benign neoplasm of colon	94 (0.37)	18 (0.22)	42 (0.53)	154 (0.37)	153 (0.09)	<0.0001
Cholecystectomy	267 (1.05)	104 (1.28)	104 (1.31)	475 (1.15)	1911 (1.15)	0.91

*p* value: reference vs. total cases.

**Table 2:** Incidence of colorectal cancer and gynecologic cancer cohorts to reference cohort adjusted hazard ratio by age.

	Reference	Cervical Cancer		Endometrial Cancer		Ovarian Cancer	
Age	Rate	Rate	aHR (95% CI)	Rate	aHR (95% CI)	Rate	aHR (95% CI)
**CRC**							
All	1.09	1.37	1.20 (1.03–1.40) *	2.20	2.26 (1.77–2.90) ***	1.76	2.09 (1.59–2.76) ***
30–39	0.19	0.22	1.14 (0.40–3.31)	1.11	6.18 (2.11–18.1) ***	1.18	6.37 (2.71–15.0) ***
40–49	0.46	0.78	1.67 (1.13–2.48) *	1.54	3.46 (2.05–5.84) ***	1.25	2.83 (1.59–5.01) ***
50–64	1.13	1.51	1.32 (1.02–1.70) *	2.34	2.19 (1.55–3.09) ***	1.48	1.41 (0.84–2.36)
≥65	2.40	2.46	1.02 (0.81–1.29)	2.84	1.23 (0.69–2.18)	4.52	1.94 (1.23–3.08) **

Incidence rate: per 1000 person–years, aHR: adjusted for age, diabetes, hypertension, benign neoplasm of colon, anal and rectal polyp and cholecystectomy. CRC, colorectal cancer. * *p* < 0.05; ** *p* < 0.01; *** *p* < 0.001.

**Table 3:** Incidence and adjusted hazard ratio of colorectal cancer by type of treatment for patients with gynecologic cancers and reference cohort.

Treatment	N	Event	Person-Years	Incidence Rate ^++^	aHR (95% CI)	aHR (95% CI)
Control	165,808	1033	945,889	1.09	1.00	
Cervical cancer						
Non-RT/CT/surgery	3369	43	22,573	1.90	1.32 (0.97–1.79)	1.00
RT	4250	40	21,555	1.86	1.11 (0.81–1.52)	0.90 (0.59–1.40)
CT	1544	8	8904	0.90	1.08 (0.54–2.16)	0.74 (0.34–1.59)
RT/CT	7558	39	29,471	1.32	1.24 (0.90–1.71)	0.97 (0.62–1.50)
Only surgery	8649	62	57,181	1.08	1.19 (0.92–1.54)	0.83 (0.56–1.25)
Endometrial cancer						
Non-RT/CT/surgery	350	7	1823	3.84	3.38 (1.61–7.11) **	1.00
RT	1376	12	5466	2.20	2.19 (1.24–3.87) **	0.61 (0.24–1.56)
CT	826	8	2872	2.79	3.39 (1.69–6.80) ***	0.83 (0.30–2.32)
RT/CT	1055	7	3325	2.11	2.38 (1.13–5.01) *	0.61 (0.21–1.76)
Only surgery	4542	34	20,132	1.69	1.97 (1.40–2.78) ***	0.50 (0.22–1.14)
Ovarian cancer						
Non-RT/CT/surgery	84	3	4121	0.73	0.98 (0.32–3.04)	1.00
RT	51	0	261	0.00		
CT	5069	30	18,368	1.63	1.95 (1.35–2.80) ***	1.88 (0.57–6.18)
RT/CT	785	3	2545	1.18	1.45 (0.47–4.49)	1.33 (0.27–6.60)
Only surgery	1184	18	5402	3.33	3.56 (2.23–5.68) ***	3.79 (1.11–12.9) *

aHR: Adjusted for age, diabetes, hypertension, benign neoplasm of colon, anal and rectal polyp and cholecystectomy. RT, radiation therapy; CT, chemotherapy. Incidence rate ++: per 1000 person-years. * *p* < 0.05; ** *p* < 0.01; *** *p* < 0.001.

## Data Availability

Data are available from the Ministry of Health and Welfare of Taiwan by request after IRB approval. Authors are not allowed to duplicate data files.
